# Water and Beverage Consumption among a Nationally Representative Sample of Children and Adolescents in the United Arab Emirates

**DOI:** 10.3390/nu11092110

**Published:** 2019-09-05

**Authors:** Habiba I. Ali, Ayesha S. Al Dhaheri, Fadima Elmi, Shu Wen Ng, Sahar Zaghloul, Eric O. Ohuma, Husain S. Qazaq

**Affiliations:** 1 Nutrition and Health Department, College of Food and Agriculture, United Arab Emirates University, P.O. Box 15551 Al Ain, UAE; 2 Independent researcher, P.O. Box 67258 Al Ain, UAE; 3 Department of Nutrition, Gillings School of Global Public Health, Carolina Population Center, University of North Carolina at Chapel Hill, Chapel Hill, NC 27599, USA; 4 National Nutrition Institute, 11562 Cairo, Egypt; 5 Centre for Tropical Medicine and Global Health, Nuffield Department of Medicine, University of Oxford, Peter Medawar Building for Pathogen Research, South Parks Road, Oxford OX1 3SY, UK; 6 School of Human Nutrition, McGill University, Montreal, QC H3A 0G4, Canada

**Keywords:** water intake, beverage consumption, children, adolescent, United Arab Emirates

## Abstract

There are limited studies examining water consumption among individuals in hot climates. We assessed the daily total water intake from plain water, other beverages, and food in a nationally representative sample of children and adolescents aged 6–18 years in the United Arab Emirates. Total water intake was compared against the recommendations of the Institute of Medicine and the European Food Safety Authority. Sociodemographic information, 24 h dietary recall, physical activity levels, and anthropometric data were collected from 527 participants. The mean ± SE of total water intake was 1778.4 ± 33.8 mL/day. Plain drinking water was the largest contributor to total water intake (51.6%), followed by food (27.3%). Sugar-sweetened beverages constituted 13.9% of water intake. The proportion of participants who met the Institute of Medicine recommendations ranged from 15% (males aged 14–18) to 25% (children aged 6–8). The proportion of participants who met the European Food Safety Authority recommendations ranged from 31% (females aged 14–18) to 36% (males aged 14–18). The water-to-energy ratio was 1.0–1.15 L/1000 kcal, meeting recommendations. The majority of participants failed to meet water intake recommendations, highlighting the need for targeted interventions to promote increased water consumption among children and adolescents.

## 1. Introduction

Water is an essential nutrient and a major constituent of the human body. It is vital to numerous physiological functions such as thermoregulation and transport of nutrients and waste products [1]. A number of conditions can influence water needs, including ambient temperature, physical activity levels, and humidity. Aside from water deficit, water intake is also influenced by numerous factors such as culture, water availability, and sensory qualities of beverages, such as taste, color, flavor, and temperature [2]. Studies have shown that cardiovascular mortality is inversely associated with water intake [3]. Furthermore, inadequate water intake has been associated with an increased risk of developing hyperglycemia [4] and recurrent kidney stones [5]. Chronic dehydration from heat stress has been implicated in the epidemic of chronic kidney disease in Central America [6,7]. Children and adolescents are susceptible to voluntary dehydration [8,9], especially during exercise, and may not recognize the need to replace lost fluids [10,11]. Mild dehydration in children can lead to impairment in cognitive functions, such as attention and short-term memory, or in executive functions, which could be detrimental to school performance [12,13]. A loss of even 1%–2% of body water has been shown to impair the ability to perform exercise [14].

Total water intake includes bottled and tap water, water in other beverages, and water content in food. Two major international organizations that have set adequate intake recommendations for water are the US Institute of Medicine (IOM) [15] and the European Food Safety Authority (EFSA) [16]. The EFSA recommendations were derived from a combination of observed intakes in individuals with desirable urine osmolarity and desirable water volume per energy units consumed (L/1000 kcal) [16]. On the other hand, IOM recommendations were based on the median intake of U.S. National Health and Nutrition Examination Survey (NHANES) III participants [15]. Water intake recommendations for children and adolescents vary by age and sex; the IOM range is 1.7–3.3 L/day [15] and the EFSA range is 1.6–2.5 L/day [16]. However, it is possible to be adequately hydrated at levels both above and below these levels [15]. Apart from comparing water intake against these guidelines, hydration status can also be assessed by measuring the volume of water consumed per 1000 kcal, with a target ratio of around 1.0–1.5 L/1000 kcal among children [16]. This ratio takes body surface area and activity level into consideration [11].

Inadequate water intake among children and adolescents has been reported worldwide with only 15%–25% of children aged 4–13 in the US [17] and 13%–19% of 9–18 year olds in Mexico [18] meeting IOM recommendations. Similar findings were observed among 9–13 year olds in the UK [19] and France [20], with 90%–94% failing to meet EFSA recommendations. In the only study that assessed total water intake in the Middle East and North Africa (MENA) region to date, 2.2%–7.9% of Lebanese 9–13 year olds met IOM recommendations [21]. However, few studies have assessed the adequacy of total water intake among healthy children and adolescents living in hot climates, who are at greater risk of water loss through sweating. The United Arab Emirates (UAE) is located in the Arabian Gulf, one of the hottest regions in the world. Temperatures can reach 50 °C in the summer. To our knowledge, total water intake from various sources has not been investigated in the UAE to date. Sugar sweetened beverages were a major source of water among participants aged 4–13 years in the UK [19] and 14–18 years in Australia [22] and Mexico [18]. A report on the Emirati diet, which was based on data from the current study, showed that sugar-added soft drinks and fruit drinks accounted for 60%–71% of calories from beverages among individuals aged 6–18 years [23], but water intake was not reported in that study. Studies have shown that replacing sugar-sweetened beverages (SSBs) with plain water is associated with less energy intake [24]. An increasing prevalence of overweight and obesity has been reported among children and adolescents in the UAE [25,26]. Therefore, it is important to evaluate the intake of plain drinking water. It is also important to consider the contributions of water from food to total water intake, as this can vary by diet. 

## 2. Materials and Methods 

### 2.1. Study Design and Participants

The present analyses used data from a national nutrition survey conducted in 2009–2010 in the United Arab Emirates. The survey included 628 nationally representative households. Survey participants were selected using a stratified random sampling method. From each randomly selected household, one child, one adolescent, and one adult female were selected. In households with more than one child, adolescent, or adult female, one from each group was randomly selected. Detailed sample selection and the methods used to collect the data have been reported previously [23,27]. The survey sample represented all the 7 emirates of the UAE and included households in both the urban and rural settings. The response rate of the selected households was 75%. The United Arab Emirates University Faculty of Medicine (protocol no. 09/13) approved the protocol of this study. Verbal and written consent were obtained from the participants. Informed consent for minors was obtained from the father or the head of the household.

In order to meet the aims of the present study, we excluded from the national nutrition survey dataset, children and adolescents with missing data on age, sex, and water consumption and the adult females. The final sample size for the present analysis was 527, including 255 children (6–10 years) and 272 adolescents (11–18 years). 

### 2.2. Data Collection

Water intake from drinking water, food, and other beverages, and demographic characteristics were collected between October 2009 and April 2010. Detailed methods for collection of dietary, anthropometric, and physical activity data have been described in previous reports [23,27]. 

Dietary data: Dietary information was based on a single 24 h recall as described in previous reports [23,27]. Trained dietitians obtained information about food and beverage intake during the preceding 24 h period using a standardized interview procedure based on the US Department of Agriculture multiple-pass method [28]. The multiple-pass method uses multiple steps (or passes) in the interview process. It prompts respondents to remember and describe foods consumed, including types, amounts, and additions [28,29,30,31,32,33,34]. The interviewers used a food instruction booklet [35], which was specially developed and used for a national survey in Kuwait [36] and adapted for UAE. This manual was designed to standardize dietary data collection and reflect cultural eating behaviors.

Information on children’s dietary intake was collected from parents and other caregivers (e.g., siblings and housemaids) in the presence of the child. Adolescents reported their dietary intake with assistance from caregivers when needed, such as information about recipe ingredients. Food models, measuring cups, spoons, and other household measures were used to assist participants recall the amounts consumed. Reported consumption of all types of beverages (caloric and noncaloric) and information about the total amount of plain drinking water and its source (tap or bottled) was recorded. Water bottles of various sizes and cups commonly used for drinking were used to estimate water intake. As the interviews were conducted in the homes of the participants, the interviewers could ask respondents to show the cups and glasses they typically used.

Anthropometry: Height and weight were measured by trained research assistants using standardized protocols developed for the study, and body mass index (BMI) was calculated [23]. We used the International Obesity Task Force (IOTF) BMI cutoffs [37,38] for underweight, normal, overweight, and obese classifications. These cutoffs were developed based on survey data from six countries and have been used to assess childhood obesity in the UAE and other gulf countries [25,39,40]. The IOTF cutoff points are age and sex specific and were extrapolated from the adult BMI cutoffs of 18.5 kg/m^2^ for underweight, 25 kg/m^2^ for overweight, and 30 kg/m^2^ for obesity.

Physical activity: Physical activity levels were measured using the short version of the International Physical Activity Questionnaire [41]. The International Physical Activity Questionnaire has been used in numerous surveys globally, including the Arabian Gulf, however it has only been validated in 15–69 year olds [42,43,44,45]. To ensure relevance, we adapted the questionnaire to be more culturally relevant and appropriate for participants under 15 years of age. We then piloted the questionnaire with adolescents (11–18 year olds) and mothers of children (6–10 year olds). Data on walking time, moderate physical activities (including rope jumping, ice-skating in the malls or at skating centers, and bicycling at a regular pace), and vigorous physical activities (including rollerblading or fast bicycling) performed, and their duration, were collected. For children aged 6–10, parents were asked to recall the activities performed by their children during the last 7 days (during school, recreation, exercise, or sport), in the presence of the child. Analysis of physical activity followed the 2005 Guidelines for Data Processing and Analysis of the International Physical Activity Questionnaire, as previously reported [23]. 

Sociodemographic data: The determination of residence type (urban or rural) was based on the 2005/2008 UAE census. Household wealth was divided into quintiles, with higher quintiles reflecting higher household wealth, as previously described [23].

### 2.3. Data Analysis

Dietary intake data were coded and analyzed by trained dietitians using the ESHA Research Food Processor SQL and ESHAPort SQL software (v. 10.4) [46]. The ESHA food composition database was updated with nutrient composition of Kuwaiti composite dishes [47,48,49]. Recipes collected during the dietary interviews were also added to the ESHA food composition database. Water amounts in food and beverages consumed during meals and snacks at home and outside the home were recorded, and their contributions to total water intake were determined. Plain water included tap and bottled water. Beverages were categorized as sugar-sweetened (regular soda, sugar-sweetened tea and coffee, and fruit drinks), noncaloric beverages (diet soda and unsweetened tea and coffee), fruit juices, milk and milk products (includes plain milk, flavored milk, milk shake, and butter milk), or other beverages (energy drinks, malt beverages, and hot chocolate). The extent to which participants met the recommended age- and sex-specific IOM and EFSA water intake was assessed by comparing total water intake against the recommendations. The water-to-energy ratio was calculated as liters of water per 1000 kilocalories consumed. All data were subjected to multiple quality checks during the collection and analysis period by field supervisors and members of the research team.

Statistical analysis using simple linear regression was performed to assess the differences in mean intake of total water and water from the various sources across each sociodemographic characteristic (age, sex, body mass index, physical activity level, quintiles of wealth, and residential setting). This was followed by analysis of mean intakes using a multiple linear regression model with all sociodemographic characteristics (age, sex, body mass index, physical activity level, quintiles of wealth, and residential setting) as covariates, to determine which sociodemographic characteristics remain associated with variation in water intake, after controlling for other covariates that could be potential confounding factors. Differences in contributions of different sources of water to total water intake between age groups were compared using ANOVA with Bonferroni adjustment. Comparisons of water intake from various sources between male and female participants in the different age groups were conducted using independent *t*-test.

Mean total water intake was compared to the age- and sex-specific IOM and EFSA recommendations. Shortfalls in total water intake were calculated. Comparisons between proportions of male and female participants meeting IOM/EFSA recommendations were performed using the Chi-squared test. Mean water intake (L) per energy consumed (1000 Kcal) was calculated for all age and gender groups. Statistical significance was assessed at the 5% level of significance (*p*-value < 0.05). All analyses were conducted using SPSS version 24.0 (IBM SPSS, Armonk, NY, USA). 

## 3. Results

### 3.1. Sociodemographic Characteristics and Water Intake

Total daily water intakes (mL/day) from plain drinking water, beverages, and food, across various sociodemographic characteristics were compared using simple linear regression (Table 1) and multiple linear regression (Table 2). The study included 527 participants with a mean ± SE total daily water intake of 1778 ± 33.8 mL. The number of participants with missing data related to BMI, physical activity level, and quintiles of wealth were 9, 26, and 35 respectively (Table 1). Intakes of total water, plain water, and water from food were significantly higher among adolescents compared to children (Table 2). Total water intake was 419.5 mL/day (*p* < 0.001) higher, plain water intake was 287.3 mL/day (*p* < 0.001) higher, and water from food intake was 84.4 mL/day (*p* = 0.001) higher (Table 2). Compared to male participants, female participants reported significantly lower intakes of total water, water from beverages (excluding plain water), and water from food (Table 2). Total water intake was 227.9 mL/day less (*p* = 0.002), water from beverages (excluding plain water) intake was 83.8 mL/day less (*p* = 0.002), and water from food intake was 55.3 mL/day less (*p* = 0.028) (Table 2). Intakes of total water and water from food were significantly higher among obese participants compared to underweight participants (*p* = 0.020 and *p* = 0.011, respectively) using simple linear regression (Table 1). However, these findings were no longer significant after controlling for sex, age group, physical activity level, residence setting, and quintiles of wealth (Table 2).

There were no significant differences in total water intake, as well as intakes of plain water, beverages excluding plain water, and water from food, among the participants based on levels of physical activity, quintiles of household wealth, and residential setting (Table 2).

### 3.2. Mean Water Intake by Age Group

Table 3 presents mean water intake from various water sources and their contributions to total water intake by age. Participants aged 14–18 reported significantly higher intake of total water, plain water, water from sugar-sweetened beverages, and other beverages (energy drinks, malt beverages) than participants aged 9–13 and 6–8 (*p* < 0.05). Plain drinking water was the main contributor to total water intake among all age groups (48.6%–53.7%), followed by water from food (25.8%–28.7%). Sugar-sweetened beverages contributed 13.9% to total water intake and milk and milk products contributed 5.2%. Participants in all age groups consumed more bottled water than tap water (40.2%–45.2% versus 8.4%–10.1%).

### 3.3. Total Daily Water Intake from All Sources by Age Group and Sex

**Figure 1:**
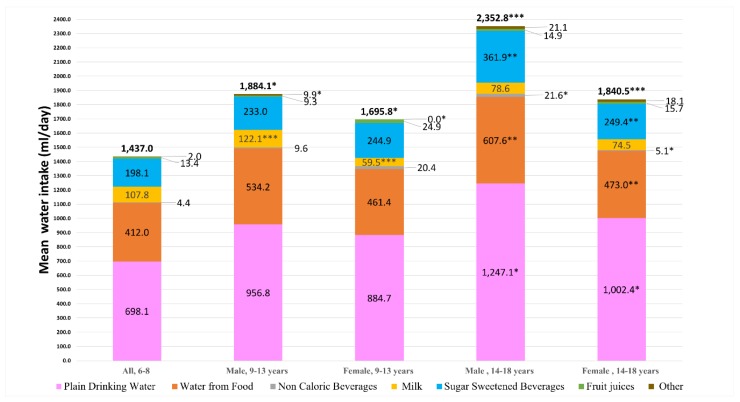
Comparison of mean water intake from various sources by age group and sex. Statistical comparisons of the means of water intake from each source of water were conducted between age and sex groups, using independent *t*-test. * *p* < 0.05; ** *p* < 0.01; *** *p* < 0.001. Sample: 6–8 years (n = 160), male 9–13 years (n = 102), female 9–13 years (n = 94), male 14–18 years (n = 80), female 14–18 (n = 91).

Figure 1 shows the mean total daily water intake from all sources by age group and sex. Among participants aged 9–13, males reported significantly higher intake of total water (1884.1 versus 1695.8 mL/day; *p* < 0.05), water from milk (122.1 versus 59.5 mL/day; *p* < 0.001), and water from other beverages (9.9 versus none; *p* < 0.05) than female participants. Male participants aged 14–18 reported significantly higher mean total water intake (*p* < 0.001), water from food (607.6 versus 473.0 mL/day; *p* < 0.01), plain drinking water (1247.1 versus 1002.4 mL/day; *p* < 0.05), water from noncaloric beverages (21.6 versus 5.1 mL/day; *p* < 0.05), and water from sugar-sweetened beverages (361.9 versus 249.4 mL/day; *p* < 0.01) than female participants.

### 3.4. Comparison of Participants’ Water Intake and IOM Recommendations

**Figure 2:**
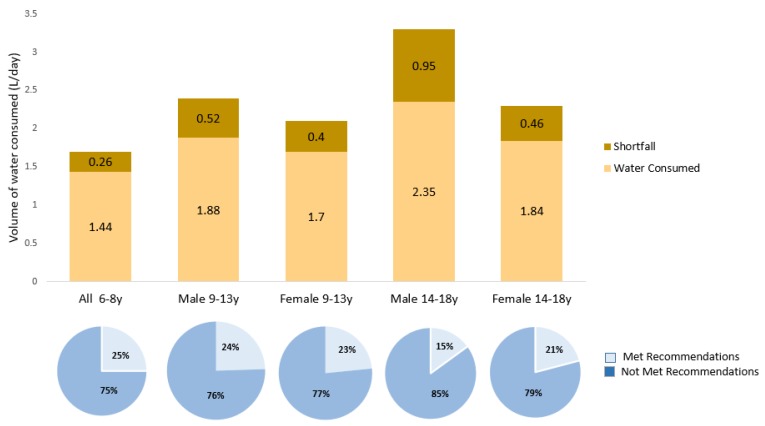
Comparison of water intake between study participants and Institute of Medicine (IOM) recommendations. Note: The total daily water intake recommendations set by IOM are 1.7 L/day for 4–8-year-olds, 2.4 L/day for 9–13 years old boys, 2.1 L/day for 9–13 years old girls, 3.3 L/day for 14–18 years old boys, and 2.3 L/day for 14–18 years old girls. Sample: 6–8 years (n = 160), male 9–13 years (n = 102), female 9–13 years (n = 94), male 14–18 years (n = 80), female 14–18 (n = 91).

Figure 2 compares the total water intake among study participants to the IOM intake recommendations. The highest proportion meeting the IOM daily water intake recommendations was observed among children aged 6–8 years (25%), while males aged 14–18 years had the lowest proportion (15%). Mean shortfalls from IOM recommendations for daily total water intake ranged from 0.26 L to 0.95 L, with participants aged 6–8 reporting the smallest shortfall and male participants aged 14–18 reporting the largest shortfall. Among participants aged 9–13, there was no significant difference between the proportion of males and females who met the IOM recommendations (24.5% versus 23.4%). Similarly, although male participants aged 14–18 reported significantly higher total water intake than female participants (2.35 versus 1.84 L/day; *p* < 0.001), there was no significant difference between the proportion of males and females who met the IOM recommendations (15.0% versus 20.9%).

### 3.5. Comparison of Participants’ Water Intake and EFSA Recommendations

**Figure 3:**
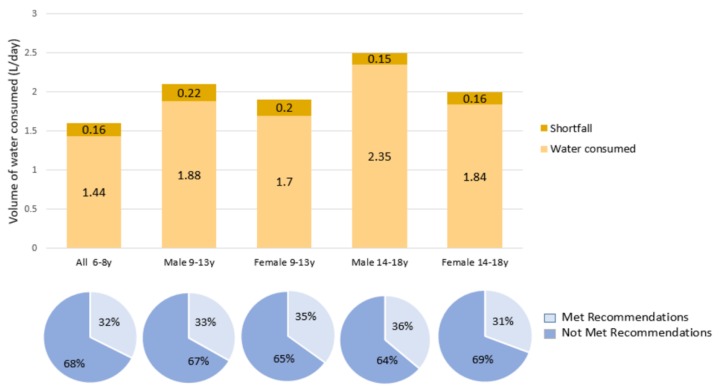
Comparison of water intake between study participants and European Food Safety Authority (EFSA) recommendations. Note: Total daily water intake recommendations set by EFSA are 1.6 L/day for children aged 4–8 years, 2.1 L/day for boys aged 9–13 years, 1.9 L/day for girls aged 9–13 years, 2.5 L/day for boys aged 14–18 years, and 2 L/day for girls aged 14–18 years. Sample: 6–8 years (n = 160), male 9–13 years (n = 102), female 9–13 years (n = 94), male 14–18 years (n = 80), female 14–18 (n = 91).

The proportions of participants meeting EFSA recommendations ranged from 31% (female participants aged 14–18) to 36% (male participants aged 14–18) (Figure 3). There were no significant sex-based differences in the proportions of participants meeting EFSA recommendations in either the 9–13 age group or the 14–18 age group. Mean shortfalls from EFSA daily water intake recommendations ranged from 0.15 L to 0.22 L, with male participants aged 9–13 reporting the largest shortfall and male participants aged 14–18 reporting the smallest shortfall.

### 3.6. Mean Water-to-Energy Ratio by Age Group and Sex

**Figure 4:**
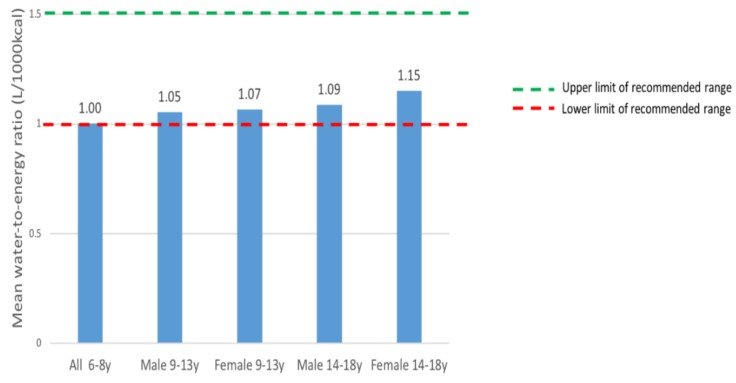
Mean water-to-energy ratio by age group and sex.

Figure 4 presents the mean water-to-energy intake ratio by age group and sex. The mean ratio reported by study participants ranged from 1.0 to 1.15, with participants aged 6–8 reporting the lowest ratio and female participants aged 14–18 reporting the highest ratio.

## 4. Discussion

In this nationally representative sample of children and adolescents, older participants (aged 14–18) reported the highest mean daily total water intake. Male participants reported higher mean total water intake and water intake from beverages, excluding plain water and food, than female participants. Age- and sex-based variations in water intake are understandable, given differences in water requirements. We did not find differences in water intake by physical activity levels, in contrast with the findings of Jomaa et al. in Lebanese children aged 4–13 [21]. This finding is concerning as adequate hydration in children is critical, especially when exercising in a hot climate [50].

Plain drinking water was the largest contributor to total water intake in all age groups. Although the UAE has stringent municipal water quality standards, bottled water was the source of over 80% of plain drinking water among participants. Our findings are consistent with the results of a recent survey among 200 parents of 3–15 year old children in the UAE [51]. In fact, the UAE was ranked the eighth largest per-capita consumer of bottled water in 2017 [52]. Tap water in the UAE is produced by desalination of seawater [53], and its unpleasant taste has been cited in a previous survey conducted in the UAE [54] as the main reason bottled water is preferred. This might explain the difference in bottled water intake between the UAE and other economically developed countries where tap water in not produced by desalination. Bottled water constituted 47% of plain water intake among children aged 4–13 in France [19] and only 16% among the same age group in the UK [18]. In the present study, water from food was the second largest contributor to total water intake (28%), consistent with the findings of Jomaa et al. in Lebanon [21].

Among Emirati children and adolescents, sugar-sweetened beverages were the most consumed beverages, excluding plain water, contributing 13.9% to total water intake. Similar findings were reported among 4–13 in the UK [19], and 14–18 in Mexico [18]. Milk and milk products were the second-most consumed beverages, excluding plain water, in our study, while milk was reported as the most consumed beverage, excluding plain water, among children aged 4–13 in the US [17] and France [20].

Previous studies have shown that regular consumption of sugar-sweetened beverages is associated with weight gain, metabolic syndrome, and type 2 diabetes [55,56]. Conducting community awareness programs to promote consumption of plain water and low-fat milk as alternatives to sugar-sweetened beverages could be considered as a public health strategy to reduce sugar-sweetened beverage intake in the UAE. The government introduced a 50% tax on soft drinks in 2017 in an attempt to reduce their consumption [57]; the effectiveness of this policy is yet to be evaluated.

We compared the daily total water intake of the study participants to two international recommendations for daily water intake, the IOM and EFSA, since there are no national daily water intake recommendations specific to the UAE population. Most participants failed to meet either IOM (75%–85%) or EFSA (64%–69%) recommendations for daily water intake. Similar findings were reported for children aged 4–13 in Lebanon [21], with 74%–97.8% failing to meet IOM recommendations and 73.5% failing to meet EFSA recommendations. Besides the study conducted in Lebanon [21], we are not aware of other studies in the Middle East or North Africa region comparing total water intake in children and adolescents with national or international guidelines. However, studies conducted in other regions [17,18,19,20] reported inadequate total water intake. Only 15%–17% of children aged 9–13 in the US [17] and 17%–19% in Mexico [18] met IOM recommendations, in contrast with the 23%–24% in our study. In our sample, 15%–21% of adolescents aged 14–18 met the IOM recommendations, while 13%–17% met recommendations in Mexico [18].

A higher proportion of participants aged 9–13 in our study (33%–35%) reported adequate water intake according to EFSA recommendations than the same age group in the UK (6%–8%) [19] and in France (7%–10%) [20]. Unlike the finding in Lebanese children aged 9–13 [21], there was no significant difference between the proportion of male and female Emirati children who met IOM recommendations. Male participants aged 14–18 in our study reported the largest shortfall in mean total water intake. The findings highlight the need for targeted interventions to increase daily water intake among children and adolescents in the UAE. One recommended strategy to increase water intake is to facilitate access to plain drinking water in schools, healthcare facilities, and public institutions. National campaigns to promote increased consumption of plain water and foods with high water content, such as fruits and vegetables, should be implemented. On a positive note, study participants reported a mean water-to-energy ratio of 1.0–1.15 L/1000 kcal, within the recommended range of 1.0–1.5 L/1000 kcal.

The major strength of this study is that it is based on a nationally representative sample and is the first study to assess the adequacy of total water intake among children and adolescents in the Arab Gulf region. Another strength of this study is that all anthropometric and dietary information was recorded using standardized measures. There are, however, a number of limitations to consider.

First, the results of this study are based on a single 24 h dietary recall, which reflects dietary intake during the previous 24 h period. Thus, it does not take into account the day-to-day variation of dietary intake. This increases the potential for over- or underestimation of water intake and should be taken into consideration when comparing these intakes against IOM and EFSA recommendations. Future national surveys should employ at least two 24 h recalls (including weekdays and weekends) to better capture usual dietary intakes of the participants. Although inaccurate fluid intake estimation among children and adolescents may occur during dietary recall [58,59], using the multiple-pass method [28] provided us with several opportunities to obtain details on food intake during the recall period, strengthening the quality of the data obtained. Second, the results of this study are based on data collected in 2009–2010. However, since this is the first and only national nutrition survey conducted in the UAE, it will be useful as a baseline for future studies. Furthermore, the data did not include summer months in the UAE (June through August), during which a much higher water intake is expected. The European Hydration Research Study [60] found that daily water intake was higher in summer than in winter. Therefore, we recommend that future studies be conducted to assess seasonal variations in water intake in the UAE. Finally, the present study did not include biomarkers of hydration, such as urine and plasma osmolality, to validate the reported total daily water intake of the participants. Nevertheless, this study makes an important contribution to the limited literature on adequacy of daily total water intake, and intake of water from various sources among Emirati children and adolescents and can serve as a baseline for future studies. Future studies, using measures that include biomarkers, are needed to assess hydration status and examine the links of hydration to health outcomes in the Emirati population.

## 5. Conclusions

The majority of the Emirati children and adolescents in the study failed to meet international water intake recommendations during non-summer months. This is of particular concern since the UAE is located in one the hottest regions in the world. Community awareness programs that promote increased plain water intake and replacing sugar-sweetened beverages with plain water, low fat milk, and fruits and vegetables are needed in the country. In addition to water consumption assessment, future studies should incorporate biochemical markers of hydration status.

## Figures and Tables

**Table 1:** Simple linear regression of water intake from drinking and the consumption of food and beverages, by sociodemographic characteristics.

Variables	n	Total Water Intake ^‡^		Plain Drinking Water ^‡^		Water from Beverages ^§,‡^		Water from Food ^‡^	
		Mean ^Ψ^ ± SEM	Coefficient (95% CI)	Mean ^Ψ^ ± SEM	Coefficient (95% CI)	Mean ^Ψ^ ± SEM	Coefficient (95% CI)	Mean ^Ψ^ ± SEM	Coefficient (95% CI)
All	527	1778.4 ± 33.79		917.3 ± 26.05		376.39 ± 12.10		484.7 ± 11.81	
Age (n = 527)									
6–10 years ^Ω^	255	1561.1 ± 35.68		776.9 ± 27.35		347.4 ± 15.92		436.8 ±15.14	
11–18 years	272	1982.1 ± 53.46	421.02 (293.07, 548.97 ***)	1049.0 ± 41.99	272.09 (172.27, 371.91 ***)	403.6 ± 17.95	56.17 (8.80, 103.54 *)	529.6 ± 17.54	92.76 (46.99, 138.53 ***)
Sex (n = 527)									
Male ^Ω^	258	1899.1 ± 50.58		966.4 ± 37.58		419.2 ± 17.31		513.5 ± 18.29	
Female	269	1662.7 ± 43.98	−236.37 (−367.71, −105.02 ***)	870.3 ± 35.97	−96.12 (−198.26, 6.03)	335.3 ± 16.57	−83.89 (−130.94, −36.84 **)	457.1 ± 14.92	−56.36 (−102.55, −10.17 *)
BMI (n = 518)									
Underweight ^Ω^	70	1592.4 ± 91.09		822.4 ± 73.65		341.2 ± 30.25		428.8 ± 26.93	
Normal	281	1781.5 ± 48.06	189.11 (−14.89, 393.12)	931.8 ± 39.66	109.40 (−48.34, 267.15)	384.7 ± 16.45	43.54 (−29.42, 116.49)	464.9 ± 13.87	36.17 (−33.76, 106.10)
Overweight	94	1821.2 ± 66.76	228.80 (−12.30, 469.90)	917.0 ± 42.76	94.59 (−91.84, 281.02)	389.5 ± 30.32	48.29 (−37.93, 134.51)	514.7 ± 30.03	85.92 (3.27, 168.56 *)
Obese	73	1894.8 ± 97.34	302.47 (47.00, 557.94 *)	993.4 ± 61.36	171.00 (−26.55, 368.54)	358.7 ± 33.69	17.57 (−73.79, 108.93)	542.7 ± 43.56	113.91 (26.33, 201.48 *)
Physical Activity level (n = 501)									
Low ^Ω^	187	1767.2 ± 64.64		889.9 ± 49.91		391.9 ± 19.44		485.4 ± 20.96	
Moderate	129	1730.7 ± 55.29	−36.45 (−212.35, 139.46)	904.5 ± 41.29	14.54 (−121.93, 151.01)	358.7 ± 26.26	−33.14 (−96.25, 29.96)	467.5 ± 24.70	−17.84 (−79.34, 43.65)
High	185	1844.8 ± 56.57	77.62 (−81.75, 236.99)	975.0 ± 44.89	85.08 (−38.56, 208.72)	377.2 ± 20.78	−14.68 (−71.86, 42.49)	492.6 ± 18.69	7.22 (−48.50, 62.94)
Quintiles of wealth (n = 492)									
1 ^Ω^	73	1721.3 ± 70.59		903.9 ± 55.12		329.5 ± 30.03		487.9 ± 30.03	
2	111	1765.5 ± 73.25	44.25 (−184.46, 272.97)	902.9 ± 55.88	−1.02 (−178.81, 176.77)	391.6 ± 25.48	61.58 (−20.25, 143.41)	471.6 ± 27.81	−16.30 (−95.84, 63.24)
3	89	1861.4 ± 108.26	140.10 (−99.57, 379.76)	965.8 ± 87.69	61.88 (−124.42, 248.19)	391.5 ± 33.90	62.05 (−23.70, 147.80)	504.1 ± 31.56	16.16 (−67.19, 99.52)
4	97	1673.3 ± 66.45	−47.94 (−283.11, 187.23)	884.2 ± 51.84	−19.73 (−202.54, 163.08)	354.2 ± 27.62	24.72 (−59.42, 108.86)	435.0 ± 21.60	−52.93 (−134.72, 28.85)
5	122	1798.9 ± 66.85	77.58 (−147.00, 302.16)	916.3 ± 49.55	12.41 (−162.17, 186.99)	388.0 ± 23.89	58.55 (−21.81, 138.91)	494.5 ± 24.44	6.62 (−71.48, 84.73)
Residence type (n = 527)									
Urban ^Ω^	282	1774.1 ± 45.36		924.6 ± 35.09		364.9 ± 16.31		484.6 ± 15.8	
Rural	245	1783.4 ± 50.65	9.32 (−123.88, 142.52)	908.9 ± 38.92	−15.69 (−118.39, 87.01)	389.6 ± 18.03	24.76 (−22.90, 72.42)	484.8 ± 17.75	0.25 (−46.30, 46.80)

CI—Confidence interval. ^Ω^ Reference category. ^§^ Excluding plain water. ^Ψ^ mL/day. ^‡^ Statistical comparisons were conducted within each sociodemographic group and within anthropometric and physical activity levels using simple linear regression. * *p* < 0.05; ** *p* < 0.01; *** *p* < 0.001.

**Table 2:** Multiple linear regression of water intake from drinking and the consumption of food and beverages, by sociodemographic characteristics (n = 464).

Variables	Total Water Intake ^‡^		Plain Drinking Water ^‡^		Water from Beverages ^§,‡^		Water from Food ^‡^	
	Coefficient	95% CI	Coefficient	95% CI	Coefficient	95% CI	Coefficient	95% CI
Age								
6–10 years ^Ω^								
11–18 years	419.48	280.77, 558.20 ***	287.34	176.87, 397.80 ***	47.75	−3.44, 98.94	84.40	35.84, 132.96 **
Sex								
Male ^Ω^								
Female	−227.94	−368.92, −86.96 **	−88.88	−201.14, 23.38	−83.77	−135.80, −31.75 **	−55.29	−104.64, −5.94 *
BMI								
Underweight ^Ω^								
Normal	110.33	−95.33, 316.00	74.16	−89.61, 237.94	35.94	−39.96, 111.84	0.23	−71.77, 72.23
Overweight	175.41	−67.53, 418.34	60.29	−133.16, 253.74	54.00	−35.65, 143.66	61.11	−23.93, 146.16
Obese	237.66	−21.74, 497.07	118.90	−87.67, 325.47	35.18	−60.55, 130.91	83.58	−7.23, 174.39
Physical Activity level								
Low ^Ω^								
Moderate	−15.93	−192.73, 160.88	31.05	−109.74, 171.84	−33.30	−98.55, 31.95	−13.68	−75.57, 48.22
High	83.47	−83.29, 250.23	98.35	−34.44, 231.14	−33.68	−95.23, 27.86	18.80	−39.58, 77.18
Quintiles of wealth								
1 ^Ω^								
2	−10.66	−242.38, 221.07	−27.63	−212.16, 156.89	50.87	−34.65, 136.38	−33.89	−115.01, 47.23
3	139.95	−104.76, 384.67	67.98	−126.89, 262.86	39.89	−50.42, 130.20	32.08	−53.59, 117.75
4	−70.24	−307.37, 166.89	−42.76	−231.59, 146.07	19.81	−67.70, 107.32	−47.29	−130.31, 35.72
5	24.16	−201.87, 250.20	−28.51	−208.51, 151.48	40.73	−42.68, 124.15	11.94	−67.19, 91.07
Residence type								
Urban ^Ω^								
Rural	−9.42	−149.26, 130.43	−26.72	−138.08, 84.64	13.02	−38.59, 64.62	4.29	−44.67, 53.25

CI—Confidence interval. ^Ω^ Reference category. ^§^ Excluding plain water. ^‡^ Statistical comparisons of mean intakes were conducted using multiple linear regression with sociodemographic, anthropometric, and physical activity level variables as covariates. * *p* < 0.05; ** *p* < 0.01; *** *p* < 0.001.

**Table 3:** Mean water intake by source and by age group.

Variable	All Participants (n = 527)		6–8 Years (n = 160)		9–13 Years (n = 196)		14–18 Years (n = 171)	
	Mean Intake (mL/day)	% of TWI	Mean Intake(mL/day)	% of TWI	Mean Intake (mL/day)	% of TWI	Mean Intake (mL/day)	% of TWI
Total water intake	1778.4 ± 33.79	100	1437.0 ± 38.96 ^a^	100	1793.8 ± 47.07 ^b^	100	2080.2 ± 73.92 ^c^	100
Plain drinking water	917.3 ± 26.05	51.6	698.1 ± 30.73 ^a^	48.6	922.2 ± 36.58 ^b^	51.4	1116.9 ± 58.20 ^c^	53.7
Bottled water	755.8 ± 26.94	42.5	577.0 ± 35.22 ^a^	40.2	741.1 ± 43.41 ^b^	41.3	940.0 ± 54.58 ^c^	45.2
Tap water	161.5 ± 20.43	9.1	121.1 ± 24.14 ^a^	8.4	181.1 ± 28.81 ^a^	10.1	176.9 ± 48.63 ^a^	8.5
Non-caloric beverages	11.0 ± 2.13	0.6	4.4 ± 2.24 ^a^	0.3	14.8 ± 4.07 ^a^	0.9	12.8 ± 4.10 ^a^	0.6
Sugar-sweetened beverages	246.9 ± 10.90	13.9	198.1 ± 15.65 ^a^	13.8	238.7 ± 18.02 ^a^	13.3	302.0 ± 21.47 ^b^	14.5
Fruit juices	15.3 ± 2.71	0.9	13.4 ± 4.35 ^a^	0.9	16.8 ± 4.67 ^a^	1	15.3 ± 4.96 ^a^	0.8
Milk	91.8 ± 5.95	5.2	107.8 ± 12.50 ^a^	7.5	92.1 ± 8.99 ^a^	5.2	76.4 ± 9.58 ^a^	3.7
Other	8.9 ± 1.97	0.5	2.0 ± 1.52 ^a^	0.2	5.2 ± 2.05 ^a^	0.3	19.5 ± 5.33 ^b^	0.9
Food	484.7 ± 11.81	27.3	412.0 ± 16.57 ^a^	28.7	499.3 ± 19.72 ^b^	27.9	536.0 ± 23.06 ^b^	25.8

Data are means ± standard error of the mean. TWI—Total water intake. ^a,b,c^ Statistical comparisons were conducted between age groups for the means of water intake from each source using analysis of variance with Bonferroni adjustment. Differing superscript letters indicate significant differences (*p* < 0.05). Sugar-sweetened beverages include regular soda, sugar-sweetened tea and coffee, and fruit drinks. Noncaloric beverages include diet soda and unsweetened tea and coffee. The category “Other” includes energy drinks, malt beverages, and hot chocolate.
